# HABIT—an early phase study to explore an oral health intervention delivered by health visitors to parents with young children aged 9–12 months: study protocol

**DOI:** 10.1186/s40814-018-0261-0

**Published:** 2018-03-27

**Authors:** Ieva Eskyte, Kara Gray-Burrows, Jenny Owen, Bianca Sykes-Muskett, Tim Zoltie, Susanne Gill, Victoria Smith, Rosemary McEachan, Zoe Marshman, Robert West, Sue Pavitt, Peter Day

**Affiliations:** 1 School of Dentistry, University of Leeds, Leeds, LS2 9LU UK; 2 Highfield Health Visiting Team, Highfield Health Centre, Leeds, BD4 9QA UK; 3 Allerton Health Visiting Team, Allerton Health Centre, Bradford, BD15 7NJ UK; 4 Born in Bradford, Bradford Institute for Health Research, Bradford, BD9 6RJ UK; 5 School of Clinical Dentistry, University of Sheffield, Sheffield, S10 2TA UK; 6 Leeds Institute of Health Sciences, University of Leeds, Leeds, LS2 9LU UK; 7 Dental Translational and Clinical Research Unit, School of Dentistry, University of Leeds, Leeds, LS2 9LU UK

**Keywords:** Parental supervised brushing, Caries, Children aged 9–12 months, Parents, Health visitors, Diet, Oral health advice, HABIT intervention, Behaviour change

## Abstract

**Background:**

Parental supervised brushing (PSB) when initiated in infancy can lead to long-term protective home-based oral health habits thereby reducing the risk of dental caries. However, PSB is a complex behaviour with many barriers reported by parents hindering its effective implementation. Within the UK, oral health advice is delivered universally to parents by health visitors and their wider teams when children are aged between 9 and 12 months. Nevertheless, there is no standardised intervention or training upon which health visitors can base this advice, and they often lack the specialised knowledge needed to help parents overcome barriers to performing PSB and limiting sugary foods and drinks.

Working with health visitors and parents of children aged 9–24 months, we have co-designed oral health training and resources (Health Visitors delivering Advice in Britain on Infant Toothbrushing (HABIT) intervention) to be used by health visitors and their wider teams when providing parents of children aged 9–12 months with oral health advice.

The aim of the study is to explore the acceptability of the HABIT intervention to parents and health visitors, to examine the mechanism of action and develop suitable objective measures of PSB.

**Methods/design:**

Six health visitors working in a deprived city in the UK will be provided with training on how to use the HABIT intervention. Health visitors will then each deliver the intervention to five parents of children aged 9–12 months. The research team will collect measures of PSB and dietary behaviours before and at 2 weeks and 3 months after the HABIT intervention. Acceptability of the HABIT intervention to health visitors will be explored through semi-structured diaries completed after each visit and a focus group discussion after delivery to all parents. Acceptability of the HABIT intervention and mechanism of action will be explored briefly during each home visit with parents and in greater details in 20–25 qualitative interviews after the completion of data collection. The utility of three objective measures of PSB will be compared with each other and with parental-self reports.

**Discussion:**

This study will provide essential information to inform the design of a definitive cluster randomised controlled trial.

**Trial registration:**

There is no database for early phase studies such as ours.

## Background

Dental caries (tooth decay) is the most prevalent preventable childhood disease and a major public health priority [1, 2]. In England, 12% of 3-year-olds and 25% of 5-year-olds are affected by caries, with figures rising to 17 and 37% for children living in deprived parts of Yorkshire [3].

Caries has a significant effect on a child, their wider family and society. In the short term, a child whose teeth are affected by caries is likely to experience pain and discomfort [4], as well as dietary changes [5, 6], and in the long term, caries may have a negative impact on speech development [7], overall health [8], quality of life [9, 10], self-esteem and social confidence [9, 11]. It also has a wider societal impact on school readiness, attendance and educational outcome [12, 13]. The cost of managing dental caries in children is substantial and accounts for a significant proportion of the £3.4 billion annual spend on NHS dentistry [14]. Caries is the most common reason for young children (over 30,000 children) to be admitted to hospital for dental care under general anaesthetic with this alone costing the NHS approximately £36 million a year [15].

Since 2012, local authorities have had a statutory requirement to commission community-based oral health promotion programmes as well as the Health Child programme. Caries prevalence in 5-year-olds is included as a key priority in the Public Health Outcomes Framework [1]. Recent NICE [16] and Public Health England [15] guidance to local authorities has recommended early-life interventions to prevent caries. One of the opportunities through which parents may engage with oral health advice is via health visitors [17, 18]. In England, health visitors and their wider teams carry out universal home visits to families when children are aged 9–12 months. Many topics are discussed at this visit including nutrition and obesity prevention, child development (including speech, language and communication), safety and oral health [19]. In some localities, these visits include the provision of a tube of fluoride toothpaste and a toothbrush. While public health guidance [14, 19] advocates the benefits of improving home-based oral health behaviours at these visits, they also identify the limited evidence of the effectiveness of these oral health conversations. Furthermore, while data from the UK is scarce, studies conducted in the US suggest that often health visitors themselves lack knowledge about oral health and diet, and skills in how to support parents to adopt protective home-based oral health behaviours for their child [20]. Limited and often absent provision of resources and training prevents health visitors from effectively communicating oral health messages [20]. Therefore, it is not surprising that health visitors often lack confidence, which prevents them from engaging more actively in oral health promotion, fulfilling their professional role [17] and shaping the foundation for children’s good oral health.

A key home-based protective oral health behaviour is toothbrushing. National guidance, for children aged 0–3 years old, recommends twice daily parental supervised brushing (PSB) with a smear of fluoride toothpaste (at least 1000 ppm) initiated from the eruption of the first tooth (around 6 months old) [21]. We will use the term parental supervised brushing (PSB) to summarise this collection of behaviours. PSB is a highly effective evidence-based approach and, where adopted, dramatically reduces early childhood caries by approximately 30% with these benefits maintained into adulthood [22–24]. Toothbrushing practices are predominantly clustered within family-based traditions with additional wider cultural influences [25]. Establishing this routine behaviour is best inculcated in infancy by the parents [25]; it is a life skill which if initiated in infancy and becomes habitual is a strong predictor for future oral health [23]. However, the adoption of PSB frequently fails [24] as it is a complex dyadic behaviour with many barriers to adoption [26, 27]. Previous interventions to encourage PSB have been simplistic and failed to recognise the complexity of this behaviour [27]. A lack of PSB is one of the reasons why caries prevalence in children and children remains unacceptably high [24].

A key focus of the Health Visitors delivering Advice in Britain on Infant Toothbrushing (HABIT) interventions is improving PSB in infancy. Advice around limiting sugary foods and drinks is also included. The HABIT intervention is underpinned by a logic model [28]. This describes what barriers to PSB need to be addressed and how this will lead to changes in motivation to undertake PSB and then an increase in PSB being undertaken. This increase in PSB leads to a reduction in early childhood caries. Whilst there are robust measures of dental caries, these require long-term follow-up (a minimum of 3 years) and are consequently more expensive [29–31]. While short-term parental-self reports of PSB exist, these are at high risk of social desirability bias. The size of this bias and the lack of objective proxy measures that robustly characterise PSB behaviour is a key evidence gap that will be addressed in this study.

### HABIT resources for an oral health intervention

Our multi-disciplinary research team together with health visiting teams and parents of children aged 9–24 months have collaborated to co-design training and materials to support oral health discussions between parents and health visitors with the aim of maximising the uptake of appropriate home-based oral health behaviours, including PSB adoption. We have called the intervention HABIT and it has been developed using the following methodology. Using professional contacts, we have collected examples of materials that have been already developed for health visitors to support their oral health conversations across England.^1^ We have then discussed these materials with parents and health visitors in a series of focus groups and interviews to identify which are most likely to lead parents to adopt appropriate oral health behaviours. These conversations have informed the development of the HABIT intervention in conjunction with our extensive research [26, 28, 32] which has followed the complex intervention development framework laid out by the MRC [33]. The intervention consists of two packages: (A) training for health visitors to deliver the HABIT intervention and (B) HABIT resources for parents.

The training for health visitors covers areas such as general oral health messages and knowledge related to toothbrushing and diet, as well as an introduction to additionally available oral health resources. We intend to use already available resources which comply with national guidance [21] such as SOAP videos (http://www.soap.media/our-courses/letstalkaboutteeth/) and the NHS e-learning package http://www.e-lfh.org.uk/programmes/healthy-school-child/. The 1-day training will also include training around how to effectively use HABIT resources to enable behaviour change conversations with parents.

The HABIT resources for parents aims to support them to adopt and maintain good oral health practices for their children and to tackle barriers that prevent them from achieving this goal. The provided resources will focus on aspects such as ‘Why is oral health important?’, ‘How to adopt protective home-based oral health behaviours?’ and ‘When to start these oral-health behaviours?’ The HABIT intervention will include short video vignettes, provision of a toothbrush and toothpaste and simple advice sheets on issues such as oral health knowledge and skills, managing children’s behaviour and the wider social environment as well as, knowledge about diet. The video vignettes include a mixture of parent stories and professional advice.

### Aims of study

This study, through working with health visitors and parents of children aged 9–12 months, aims (i) to explore the acceptability of the HABIT intervention to health visitors and parents of children, (ii) to examine the mechanism of action of the HABIT intervention on PSB adoption and maintenance and (iii) to develop a suitable objective measure(s) of PSB adoption.

Using a mixed-methods approach, it will specifically:

- Explore how the HABIT oral health intervention works in practice and how it influences behaviour change
- Identify intervention mechanisms that are most likely to lead to the adoption of PSB within the daily routine
- Establish the acceptability of the HABIT oral health intervention to parents and health visitors
- Develop and correlate different objective measures of toothbrushing with parental self-reports of PSB

## Methods/design

This mixed-methods study will involve two participant groups: (A) health visitors (*n* = 6) and (B) parents of children aged 9–12 months (*n* = 30) to allow the objectives to be achieved and to capture the perspectives of all relevant stakeholders. For each participant group the design, procedure and approach to data collection and analysis will be described.

### Acceptability of the HABIT resources for health visitors

#### Design of the study

This part of the study will test the acceptability of the HABIT resources for health visitors undertaking a universal home visit to families of children aged 9–12 months. Firstly, recruited health visitors will be trained to deliver the HABIT intervention. This training will include an update on oral health to ensure all health visitors provide uniform evidence-based advice [21], training on how to use the HABIT resources and discussions on how to engage with parents in a behaviour change conversation. After receiving the training, each of the health visitors will undertake an oral-health conversation using the HABIT resources to five parents of children aged 9–12 months during the universal home visit. After each home visit, the health visitors will complete a semi-structured diary where they will reflect on parents’ child toothbrushing habits and their reaction and response to the HABIT resources as well as their own experience when using these resources.

Once all six health visitors have delivered the intervention, they will participate in a focus group. A topic guide will be used as the framework for the discussion and will shed light on the received training and experience of using the HABIT intervention for parents, its strengths and weaknesses as well as whether it fits everyday professional practice. Focus group discussion will provide deeper and broader insight into why certain opinions, positions and perspectives are held [34] and the issues that may prevent or facilitate implementation of the intervention [35]. The topic guide has been developed based on Ayala et al.’s [35] definition of acceptability and Gray-Burrows et al.’s [28] model for intervention mapping to develop a home-based PBS intervention for young children. The two frameworks will enable the exploration of the acceptability of the HABIT intervention as well as key barriers and facilitators (e.g. knowledge, skills, confidence and attitudes), which may influence health visitors’ experience and intentions to use the resources. The focus group will be digitally recorded and professionally transcribed after the event. The recording will be deleted after accuracy of the transcription and recording is checked. All identifying information will be removed from the transcripts to ensure anonymity.

#### Setting and recruitment

The research participants will be recruited from the health visiting teams employed by the local community NHS trust. Participants will be purposefully recruited to include health visitors and their wider teams who are about to deliver a universal home visit to families of children aged 9–12 months—the target age group for the HABIT intervention.

The health visiting leads (SG & VS) for this project will identify health visitors and their wider teams who deliver regular universal home visits to parents of children aged 9–12 months according to the inclusion/exclusion criteria described below. The identified health visitors and their wider teams will be emailed through their local senior manager, with the covering letter, participant information sheet and consent form. Interested participants will be contacted by the NHS trust Clinical Studies Officer, who will explain the study and obtain written consent.

#### Sample size

Six health visitors will be involved in the study in order to assess variation in that role. Although much of the assessment will be qualitative, there will be sufficient data to estimate an intra-class correlation coefficient (ICC) and confidence intervals. This ICC estimate is likely to be imprecise and will need to be supported by other similar or future studies.

#### Type of participants

The following inclusion and exclusion criteria will be adopted:

*Inclusion criteria*:

- Health visitors/wider members of health visiting teams who are about to deliver a universal home visit to parents of children aged 9–12 months in the local community

*Exclusion criteria*:

- Health visitors/wider members of health visiting teams who infrequently deliver universal home visit at 9–12 months age

#### Data collection

Diaries will be sent out to the recruited health visitors’ work email addresses. Diaries will be set up with password protection, thus enabling the health visitor to securely return the diary to the HABIT research team once they have populated their thoughts. It will take around 5–7 min to complete the diary.

The focus group will be conducted at a time and place most convenient for all six recruited health visitors and wider members of health visiting teams. The focus group will be preferably arranged during office hours and in a convenient location. The focus group will be recorded using a digital sound recorder and will last 60–75 min.

#### Data analysis

Diary and focus group data will be analysed using framework analysis based on Ayala et al. definition of acceptability [35] and Gray-Burrows et al.’s [28] model for intervention mapping to develop a home-based parental-supervised toothbrushing intervention for young children. Transcripts will be coded using an inductive approach, and thematic analysis [36, 37] will be carried out. The data will be managed with the computer software programme QSR NVivo 10.

### Acceptability of the HABIT resources for parents

#### Design of the study

This part of the study will use mixed methods to test acceptability of the HABIT resources for parents of children aged 9–12 months. Prior to the mandatory 9–12-month Child Health Review by the HABIT-trained health visitor, the HABIT research team will visit parents who have consented to take part in the study and collect baseline PSB data. Parents will then receive a home visit by the trained health visitor who will use the HABIT resource package for parents. At 2 weeks and 3 months following the HABIT intervention, the research team will undertake further home-based data collection around effectiveness of current toothbrushing, duration and parent/children (dyad) interaction during toothbrushing, and toothbrushing activity. This will enable changes in PSB practices and attitudes to be monitored. Home-based qualitative semi-structured interviews with parents will then be undertaken to explore deeper and wider structures behind barriers and facilitators that influence parents’ behaviour, and acceptability of the provided intervention and HABIT resources for parents.

#### Setting and recruitment

The research participants will be recruited purposefully from the health visiting 9–12-month waiting list. The sample will be recruited to involve parents of children aged 9–12 months living within the local community with a range of different socio-economic and ethnic minority groups. A total of 30 parents will be recruited.

Suitable parents will be identified by a Clinical Systems Specialist with the Health Visiting team who will run a report for each of the six recruited, HABIT-trained, health visitors. The report will identify the next 20 families that are due to receive a 9–12-month home visit for each of the six HABIT-trained health visitors. An invitation letter, participant information sheet and consent form will accompany a reminder notice that is normally posted to parents prior to the home visit. Participants will continue to be recruited until five parents of each of the six recruited health visitors consent to take part in the study. If a parent withdraws from the study before the health visitors attend the focus group, a new parent will be recruited and will undergo the same research process as other participants. However, if a parent decides to stop participation after the focus group with health visitors has taken place, new parents will not be recruited. Interested parents will be consented by the NHS trust Clinical Studies Officer. Recruitment will be restricted to parents of children aged 9–12 months due to the nature of the HABIT resources that specifically target this age group.

#### Sample size

The sample size of 30 participants (e.g. five parents from each of the six HABIT-trained health visitors) will be selected so that the key parameters for a trial could be determined including acceptability of the HABIT resources, exploration of intervention mechanism and acceptability of data collection in the home setting.

#### Type of participants

The following inclusion and exclusion criteria will be adopted:

*Inclusion criteria*:

- Parents of children aged 9–12 months who are about to receive a universal home visit by the HABIT-trained health visitor
- Parents of children age 9–12 months who live in the local community
- Parents who meet the above inclusion criteria and whose children have at least one erupted tooth

*Exclusion criteria*:

- The opposite of those described above

#### Data collection

Data will be collected about the following variables: sociodemographic characteristics, effectiveness of current toothbrushing, duration and parent/child interaction during toothbrushing, toothbrushing activity, self-reported toothbrushing and dietary behaviours, acceptability of the HABIT intervention and qualitative enquiry to explore mechanism of action.

Data collection with parents will consist of at least three rounds:

1. First round—*Baseline data collection*. In the home setting, baseline PSB data will be collected before the universal home visit provided by the HABIT-trained health visitor. A researcher will ask validated questions of parents about their PSB habits, e.g. self-reported PSB [24] including questions about brushing frequency, use of fluoride toothpaste, parental involvement, and age of initiation. Three different proxy objective measures of PSB will be collected:

- Effectiveness of current toothbrushing—children pre-brushing plaque levels on the buccal surface of each erupted primary tooth will be quantified using an established index [38];
- Duration and parent/child (dyad) interaction during toothbrushing—the researcher will film the dyad toothbrushing and this will be subsequently evaluated by the HABIT research team using an established PSB index [39];
- Toothbrushing activity—parents will be provided with a regular children toothbrush in conjunction with a Magic Timer app for their phone/tablet which monitors frequency and duration of children brushing. Magic Timer app is freely available on the Internet for members of the public. For parents who do not have or are unable to use devices providing access to the app, a hard copy of the Magic Timer diary with aspects of the Magic Timer app will be provided.

For measuring plaque level, a food colouring agent will be used and then wiped away. In cases where children are allergic to a food colouring agent or parents refuse these to be used, pre-brushing plaque levels will not be measured. The activity will be carried out either at the parent’s home setting or a health care setting where they normally see a health visitor. The baseline data collection will approximately take 30–45 min.

Plaque level data and filmed materials will be accessible only for the HABIT research team and any of the information that could identify them or a child will not be available outside the HABIT research team. Only the videos for which parents provide written consent to be used for training purposes will be used by the HABIT research team when delivering training for health visitors or for other professional activities.

Sociodemographic data will be collect about parents’ ethnicity, education, employment status and experience, and family finances. We will also collect dietary data on type and frequency of foods and drinks parents give to their children on everyday basis, breastfeeding, bedtime routine, and drinking and dummy/thumb sucking practices [40].

*Intervention delivery*. Dyads (parent/child) will then receive their home visit where the HABIT intervention will be delivered by the trained health visitor as part of their mandatory 9–12-month Healthy Child review.

2. Second round*—2-week data collection*. At 2 weeks following the intervention delivered by the trained health visitor, further self-reported and objective measures of PSB (see a-c) and self-reported dietary data will be collected following the protocols identified.
3. Third round—*3-month data collection*. Identically to the second round, at 3 months after the intervention, further self-reported and objective measures of PSB (see a-c) and self-reported dietary data will be collected following the protocols identified. This measurement schedule is shaped by the time taken for habits to become established [41], e.g. PSB adoption.
4. Fourth round—*qualitative interviews*. Parents who took part in one or more rounds of the study will be invited to attend a qualitative interview. A topic guide will be used as the framework for the discussion and will shape the conversation around the experience of receiving oral health advice by the trained health visitor, the HABIT information package for parents and their usability in everyday child toothbrushing practices, strengths and weakness of the resources and potential behaviour changes after the intervention. The parents will be also invited to discuss acceptability of the data collection procedures and assessment. Qualitative face-to-face interviews will provide deeper and broader insight into how cultural, interactional, contextual and situational factors shape parents’ position toward and experiences of child oral health and toothbrushing as well as enabling us to detect and link social structures and processes that affect these positions and experiences [42]. The topic guide has been developed based on Ayala et al.’s [35] definition of acceptability and Gray-Burrows et al.’s [28] model for intervention mapping to develop a home-based parental-supervised toothbrushing intervention for young children. The two frameworks will enable the exploration of the acceptability of the HABIT resources as well as key barriers and facilitators (e.g. knowledge, skills, confidence and attitudes) which may influence parents’ experience and intentions to use the resources and initiate behaviour change. The interviews will take place either in the parent’s home or a public location convenient to a participant such as a café or a coffee shop where they feel comfortable talking about their infant’s oral health. The interview will last approximately 45–60 min and will be digitally recorded and professionally transcribed after the event. The recording will be deleted after accuracy of the transcription and recording is checked. All identifying information will be removed from the transcripts to ensure anonymity.

#### Data analysis

**Fig. 1:**
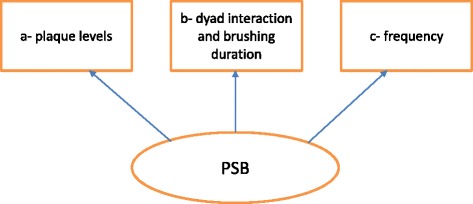
The measurement model

This study will produce three objective measures (a-c) which aim to quantify the underlying latent concept of PSB. These will be combined with a ‘measurement model’ (see Fig. 1) as it is referred within the structural equation modelling literature. It is anticipated that as the quality of PSB increases, the three objective measures will increase. The latent variable PSB will capture those components which co-vary. That is, PSB will quantify the covariance of the objective measures.

**Fig. 2:**
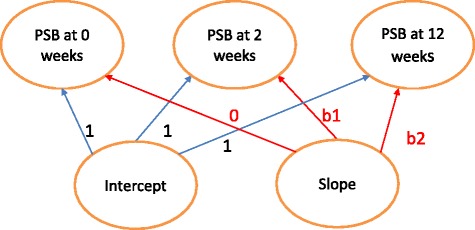
The growth model

Since PSB will be captured at three different times (0, 2 and 12 weeks), a growth model (see Fig. 2) can be fitted with a baseline value and a slope value.

Factor loadings will be available from the measurement model. By generating a standardised model where the variance of each objective measure is scaled to unity, the associate standardised factor loadings will effectively rank the measures according to the strength of their contributions to PSB. These will be taken as the quantitative assessment for each measure.

The qualitative data will be analysed in a similar way to that described earlier. The decision as to the most appropriate measure to use in a future trial will draw upon both quantitative and qualitative evidence and will be pragmatic.

## Discussion

This study protocol is designed to evaluate the acceptability of the HABIT resources for the oral health intervention delivered by health visitors during a mandatory visit to parents of children aged 9–12 months. The findings will provide valuable information regarding knowledge and skills of health visitors, their wider teams, and parents of taking care of child’s oral health and toothbrushing. It will also provide insight into motivation and external factors (social, cultural, societal, interactional, contextual, etc.) behind certain behaviours, as well as support and assistant needed for the two stakeholder groups to engage in and maintain healthy oral behaviours. Finally, the study will evaluate whether and how HABIT intervention shape oral health behaviour changes and establish the utility of different objective measures of toothbrushing with parental self-reports of PSB.

Children living in deprived families are more likely to develop caries [3]. Ethnicity and child’s decay status may also influence PSB [43]. Respectively, involvement of participants from different backgrounds is essential for ensuring the validity and reliability of the collected data. This study will seek to involve parents from different socio-economic and ethnic minority groups.

In terms of participant comfort, the study does not seek to reveal any sensitive issues, and it is not anticipated that the participants will feel distressed during the course of the research. However, some participants may find the discussions difficult or embarrassing. For instance, some parents may feel embarrassed by their current toothbrushing habits or lack of skills, and health visitors and members of their wider teams may feel uncomfortable if they think they do not have enough knowledge about the subject. In order to minimise potential and similar risks, the research team will identify health visitors who are not involved in the study and have agency and expertise to assist those who need support.

Parents’ participation in the study requires them to be involved in the research activities outlined throughout 3 to 4 months. These include ensuring that parents feel comfortable with the research team and activities, and data collection meetings are organised when most convenient to a participant, in order to maintain enthusiasm to ensure progress and momentum of the study [22]. As a thank you for their time and participation, after each data collection visit, parents will be provided with a £10 Love2Shop voucher.

The study findings will be widely disseminated via academic, professional and public venues. With regard to academic data dissemination, research findings will be published in a peer-reviewed health care journal and as conference abstracts and presentations. In terms of data distribution to professionals, at the end of the project, an event for health visitors, public health professionals and commissioners will be organised that will provide a platform to engage in further discussion with the professionals. A wider programme of dissemination will involve parents and the public. The findings will be disseminated back to this group of participants in a lay report and a video vignette that will be developed together with Better Start Bradford and community members who will advise on the most appropriate method of dissemination to the local community. Furthermore, research findings to a wider community will be disseminated via Facebook, Twitter, Mumsnet, and Dadsnet, as well as participating in public forums such as the Born in Bradford family festival, and utilising the Born in Bradford parent governors group.

## Conclusions

This early phase study will ensure the support for PSB is suitable for health visitors to deliver and acceptable to parents. It will identify the most appropriate objective measure/s of PSB. It will enable an estimate of intra-class correlation coefficient (ICC) and confidence intervals for designing the definitive trial. The results will be used to design a larger study to test whether the PSB intervention can prevent decay (effectiveness) and save the NHS money.

## Abbreviations

CRN: Clinical Research Network
HABIT: Health Visitors delivering Advice in Britain on Infant Toothbrushing
ICC: Intra-class correlation coefficient
NHS: National Health Service
NIHR: National Institute for Health Research
PSB: Parental supervised brushing
SG: Susanne Gill
VS: Victoria Smith

## Footnotes

1 Derbyshire Community Health Services NHS Foundation Trust; Blackpool Centre for Early Child Development; Nottinghamshire Healthcare NHS Foundation Trust; Public Health and Health Improvement Leicester City Council; Public Health and Adult Social care, City of Stoke-on-Trent; Public Health England; Bradford District Care NHS Foundation Trust Community Dental Service; Ingrid Perry, Co-founder and Secretary of Teeth Team
